# Crystal structure of (–)-(*R*,*E*)-3-(1,3-benzodioxol-5-yl)-5-[(4*S*,5*R*)-5-hy­droxy­methyl-2,2-dimethyl-1,3-dioxolan-4-yl]-*N*,*N*-di­methyl­pent-4-enamide

**DOI:** 10.1107/S2056989018007132

**Published:** 2018-05-18

**Authors:** Takeshi Oishi, Koki Ishii, Mizuki Ishibashi, Takaaki Sato, Noritaka Chida

**Affiliations:** a School of Medicine, Keio University, Hiyoshi 4-1-1, Kohoku-ku, Yokohama 223-8521, Japan; b Department of Applied Chemistry, Faculty of Science and Technology, Keio University, Hiyoshi 3-14-1, Kohoku-ku, Yokohama 223-8522, Japan

**Keywords:** crystal structure, 1,3-dioxolane, 1,3-benzodioxole, amide, hy­droxy group, hydrogen bond

## Abstract

In the title compound, C_20_H_27_NO_6_, the amide moiety is essentially planar, with a maximum deviation of 0.073 (3) Å, and one of the *N*-methyl groups shows rotational disorder. The five-membered 1,3-dioxolane ring adopts an envelope form, with the C atom bonded to the olefin side chain as the flap, which deviates from the mean plane through the other four atoms by 0.564 (7) Å. The 1,3-dioxole ring fused to the benzene ring adopts a flattened envelope form, with the C atom between the two O atoms as the flap, which deviates from the mean plane through the other four atoms by 0.215 (7) Å. The C—C=C—C olefin moiety is essentially planar and makes a dihedral angle of 87.1 (3)° with the benzene ring. An intra­molecular O—H⋯O hydrogen bond supports the mol­ecular conformation, enclosing an *S*(11) graph-set motif. In the crystal, inter­molecular C—H⋯O hydrogen bonding links the mol­ecules into a tape running along the *b* axis. Furthermore, other weak C—H⋯O hydrogen bonds and a C—H⋯π inter­action connect the tapes into a sheet structure parallel to (100).

## Abstract 2

In the title compound, 1,3-dioxolane and 1,3-dioxole rings adopt envelope forms, while amide moiety and benzene ring are essentially planar. An intra­molecular O—H⋯O hydrogen bond supports the mol­ecular conformation, and inter­molecular weak C—H⋯O and C—H⋯π inter­actions connect the mol­ecules into sheet structure.

## Chemical context   

Five-membered cyclic acetal is a pervasive building block in organic synthesis since it is easily prepared from an aliphatic or an aromatic 1,2-diol. These conversions are often carried out with protection of the contiguous diol (Wuts, 2014 ▸) to prevent unexpected side reactions or to reduce the polarity of the substrate, especially for carbohydrates. Although masking of the hy­droxy groups is a disadvantage in terms of crystallization, due to loss of hydrogen-bond donors, it is expected to stabilize the crystal packing in order to contribute conformational rigidity by forming the cyclic acetal (Vijayasaradhi *et al.*, 2003 ▸).
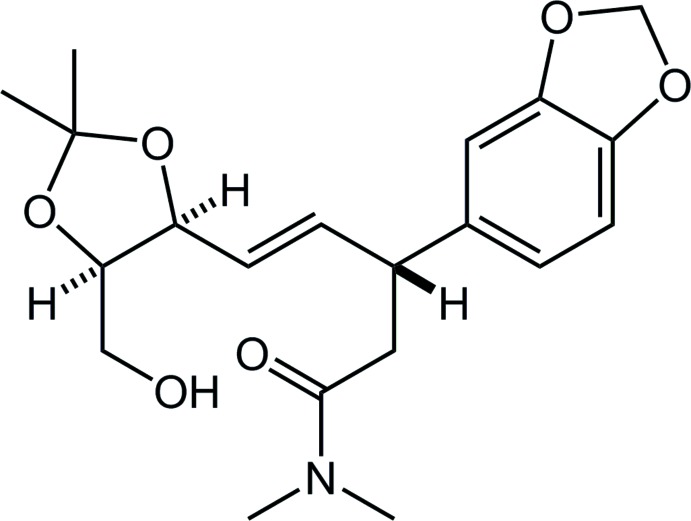


The title compound is an inter­mediate in the total synthesis of a natural alkaloid (Ishii *et al.*, 2018 ▸) possessing both 1,3-dioxolane and 1,3-benzodioxole components. The relative configurations were confirmed by the X-ray analysis as C7*R*, C10*S* and C14*R*.

## Structural commentary   

**Figure 1:**
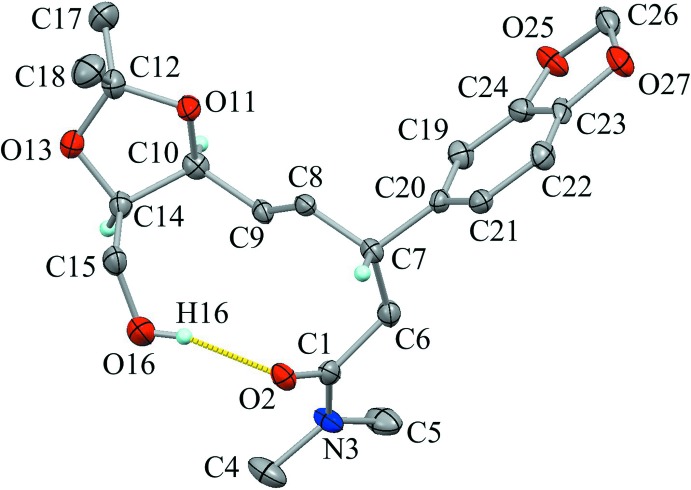
The mol­ecular structure of the title compound, with the atom labelling. Displacement ellipsoids are drawn at the 50% probability levels. A yellow dotted line indicates the intra­molecular O—H⋯O hydrogen bond. Only H atoms connected to O and chiral C atoms are shown for clarity.

The mol­ecular structure of the title compound is shown in Fig. 1 ▸. The amide moiety (C1/O2/N3/C4–C6) is essentially planar, with a maximum deviation of 0.073 (8) Å at atom C4. One of the *N*-methyl groups (C4) shows rotational disorder over two orientations, with refined occupancies of 0.54 (8) and 0.46 (8). The 1,3-dioxolane ring (C10/O11/C12/O13/C14) adopts an envelope form, with puckering parameters of *Q*(2) = 0.362 (5) Å and φ(2) = 40.6 (7)°. The flap atom C10 deviates from the mean plane through the other four atoms by 0.564 (7) Å. The 1,3-dioxole ring (C23/C24/O25/C26/O27) in benzodioxole adopts a flattened envelope form, with puckering parameter of *Q*(2) = 0.135 (5) Å and φ(2) = 326 (2)°. The flap atom C26 deviates from the mean plane through the other four atoms by 0.215 (7) Å. The olefin moiety (C7—C8=C9—C10) is essentially planar and makes a dihedral angle of 87.1 (3)° with the benzene ring (C19–C24). An intra­molecular O—H⋯O hydrogen bond (O16—H16⋯O2; Table 1 ▸) supports the mol­ecular conformation, generating an *S*(11) graph-set motif.

## Supra­molecular features   

**Figure 2:**
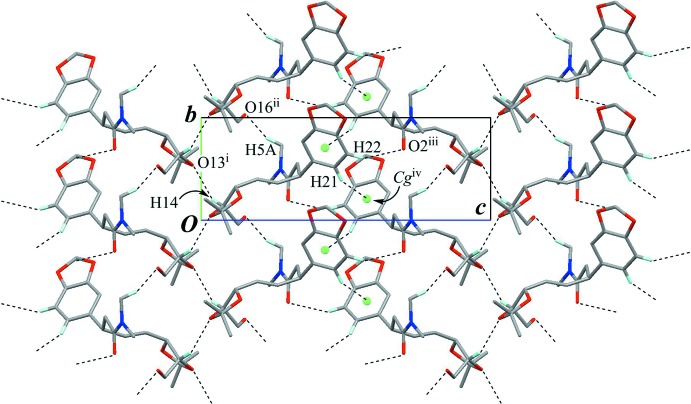
The crystal packing of the title compound, viewed down the *a* axis, showing the sheet structure parallel to (100). Black dashed lines indicate inter­molecular C—H⋯O and C—H⋯π inter­actions. *Cg* (green sphere) is the centroid of the C19–C24 benzene ring. Only H atoms involved in the above inter­actions are shown for clarity. [Symmetry codes: (i) −*x* + 1, *y* + 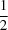, −*z*; (ii) *x*, *y* + 1, *z*; (iii) −*x* + 1, *y* + 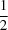, −*z* + 1; (iv) −*x* + 1, *y* – 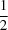, −*z* + 1.]

The crystal packing is stabilized by a C—H⋯O inter­action (C14—H14⋯O13^i^; symmetry code as in Table 1 ▸), which links the mol­ecules into a tape running along the *b* axis, with a *C*(3) graph-set motif. Furthermore, other weak C—H⋯O hydrogen bonds and a C—H⋯π inter­action (C5—H5*A*⋯O16^ii^, C22—H22⋯O2^iii^ and C21—H21⋯*Cg* ^iv^; *Cg* is the centroid of the C19–C24 benzene ring; Table 1 ▸) connect the tapes into a sheet parallel to (100) (Figs. 2 ▸ and 3 ▸).

## Database survey   

**Figure 4:**
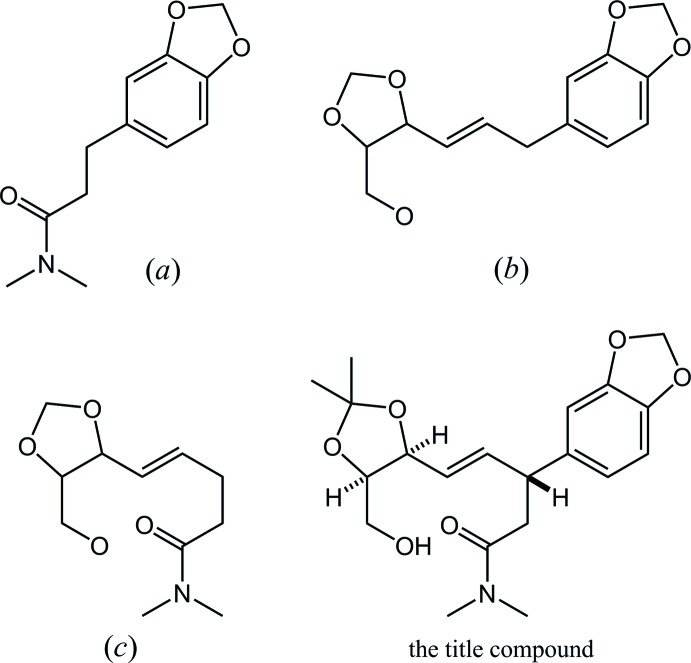
The core structures for the database survey, showing (*a*) 3-(1,3-benzodioxol-5-yl)-*N*,*N*-di­methyl­penta­namide, (*b*) 5-[3-(2,2-dimethyl-5-oxymethyl-1,3-dioxolan-4-yl)prop-2-en-1-yl]-1,3-benzodioxole and (*c*) 5-(2,2-dimethyl-5-oxymethyl-1,3-dioxolan-4-yl)-*N*,*N*-di­methyl­pent-4-en­amide.

In the Cambridge Structural Database (CSD, Version 5.39, last update February 2018; Groom *et al.*, 2016 ▸), 32 structures are registered which contain a skeleton with a combination of benzodioxole and *N*,*N*-di­methyl­amide components, (*a*), similar to the title compound (Fig. 4 ▸). These include 12 structures with no other substituent on the 1,3-benzodioxole; the 1,3-dioxole rings in eight structures adopt envelope forms similar to the title compound, while those in three structures show planar (one structure has no geometrical details in the CIF).

On the other hand, searching the CSD for a structure with a combination of benzodioxole and oxymethyl­dioxolane components, (*b*), gives two entries with refcodes YERGUX (Doyle *et al.*, 1994 ▸) and ZEMKOR (Doyle *et al.*, 1995 ▸). The forms of the 1,3-dioxoles in these two structures resemble the title compound, with the C—O—C—C torsion angles (absolute value) being 7.2 (6) and 6.6 (7)° in YERGUX, 9.3 (7) and 10.1 (7)° in ZEMKOR, and 8.4 (5) and 9.5 (5)° in the title compound. The 1,3-dioxolane rings also show a similar conformation, with the torsion angles being 24.1 (5) and 31.3 (5)° in YERGUX, 23.1 (7) and 36.8 (7)° in ZEMKOR, and 24.8 (5) and 36.0 (5)° in the title compound. No structure with a combination of oxymethyl­dioxolane and *N*,*N*-di­methyl­amide components, (*c*), has yet been reported.

## Synthesis and crystallization   

The title compound was synthesized in two steps from 3,4-*O*-iso­propyl­idene-3-d-arabino­pyran­ose (Gelas & Horton, 1975 ▸), by coupling with a known benzodioxole analogue (Rotherham & Semple, 1998 ▸) and further manipulations (Ishii *et al.*, 2018 ▸). Purification was carried out by silica-gel column chromatography, and colourless crystals were afforded from a di­chloro­methane solution under a toluene-saturated atmosphere by slow evaporation at ambient temperature (m.p. 409–410 K). [α]_D_^23^ −46.0° (*c* 1.01, CHCl_3_). HRMS (ESI) *m*/*z* calculated for C_20_H_27_NO_6_Na^+^ [*M* + Na]^+^: 400.1736; found: 400.1731.

## Refinement   

Crystal data, data collection and structure refinement details are summarized in Table 2 ▸. The absolute configuration of the title compound was determined according to the known stereochemistries of atoms C10 and C14 derived from d-arabinose. The H atoms on one of the *N*-methyl groups (C4) are disordered; they were split into two sets of positions H4*A*–*C* and H4*D*–*F*, the refined occupancies being 0.54 (8) and 0.46 (8), respectively. C-bound H atoms were positioned geometrically, with C—H = 0.95–1.00 Å, and constrained to ride on their parent atoms, with *U*_iso_(H) = 1.5*U*_eq_(C) for methyl groups or 1.2*U*_eq_(C) otherwise. The hy­droxy H atom was placed in a difference map and treated as riding, with O—H = 0.84 Å and *U*_iso_(H) = 1.5*U*_eq_(O). One problematic reflection (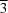,0,16) was omitted in the final refinement.

## Supplementary Material

Crystal structure: contains datablock(s) global, I. DOI: 10.1107/S2056989018007132/is5496sup1.cif

Structure factors: contains datablock(s) I. DOI: 10.1107/S2056989018007132/is5496Isup2.hkl

Supporting information file. DOI: 10.1107/S2056989018007132/is5496Isup3.cml

CCDC reference: 1842600

Additional supporting information:  crystallographic information; 3D view; checkCIF report

## Figures and Tables

**Figure 3:**
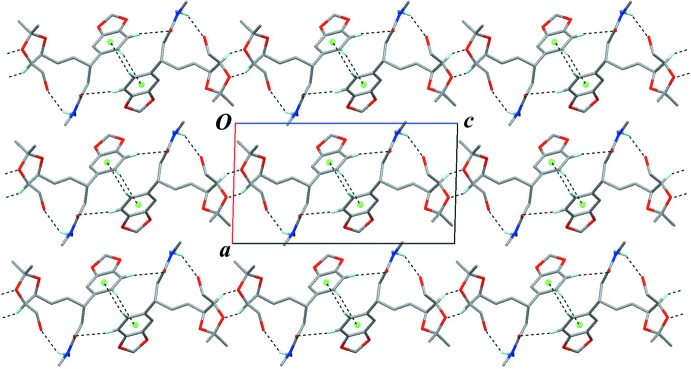
The crystal packing of the title compound, viewed down the *b* axis, showing layered sheet structures parallel to (100). Black dashed lines indicate inter­molecular C—H⋯O and C—H⋯π inter­actions. *Cg* (green sphere) is the centroid of the C19–C24 benzene ring. Only H atoms involved in the above inter­actions are shown for clarity.

**Table 1:** Hydrogen-bond geometry (Å, °) *Cg* is the centroid of the C19–C24 benzene ring.

*D*—H⋯*A*	*D*—H	H⋯*A*	*D*⋯*A*	*D*—H⋯*A*
O16—H16⋯O2	0.84	1.99	2.810 (5)	166
C14—H14⋯O13^i^	1.00	2.43	3.253 (6)	139
C5—H5*A*⋯O16^ii^	0.98	2.55	3.417 (7)	147
C22—H22⋯O2^iii^	0.95	2.55	3.417 (5)	152
C21—H21⋯*Cg* ^iv^	0.95	2.98	3.794 (4)	145

Symmetry codes: (i) 
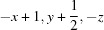
; (ii) 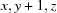; (iii) 
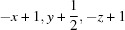
; (iv) 
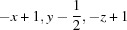
.

**Table 2:** Experimental details

Crystal data	
Chemical formula	C_20_H_27_NO_6_
*M*_r_	377.42
Crystal system, space group	Monoclinic, *P*2_1_
Temperature (K)	90
*a*, *b*, *c* (Å)	9.2538 (6), 6.0642 (4), 17.1441 (10)
β (°)	91.475 (2)
*V* (Å^3^)	961.75 (11)
*Z*	2
Radiation type	Mo *K*α
μ (mm^−1^)	0.10
Crystal size (mm)	0.27 × 0.16 × 0.10

Data collection	
Diffractometer	Bruker D8 Venture
Absorption correction	Multi-scan (*SADABS*; Bruker, 2016 ▸)
*T*_min_, *T*_max_	0.97, 0.99
No. of measured, independent and observed [*I* > 2σ(*I*)] reflections	8906, 2900, 2454
*R*_int_	0.046
(sin θ/λ)_max_ (Å^−1^)	0.596

Refinement	
*R*[*F* ^2^ > 2σ(*F* ^2^)], *wR*(*F* ^2^), *S*	0.046, 0.099, 1.07
No. of reflections	2900
No. of parameters	250
No. of restraints	1
H-atom treatment	H-atom parameters constrained
Δρ_max_, Δρ_min_ (e Å^−3^)	0.25, −0.25

Computer programs: *APEX3* (Bruker, 2016 ▸), *SAINT* (Bruker, 2016 ▸), *SHELXT2014* (Sheldrick, 2015*a* ▸), *SHELXL2014* (Sheldrick, 2015*b* ▸), *Mercury* (Macrae *et al.*, 2008 ▸), *publCIF* (Westrip, 2010 ▸) and *PLATON* (Spek, 2009 ▸).

## supplementary crystallographic information

### Crystal data

**Table:**

C_20_H_27_NO_6_	*D*_x_ = 1.303 Mg m^−^^3^
*M**_r_* = 377.42	Melting point = 409–410 K
Monoclinic, *P*2_1_	Mo *K*α radiation, λ = 0.71073 Å
*a* = 9.2538 (6) Å	Cell parameters from 3257 reflections
*b* = 6.0642 (4) Å	θ = 2.5–24.9°
*c* = 17.1441 (10) Å	µ = 0.10 mm^−^^1^
β = 91.475 (2)°	*T* = 90 K
*V* = 961.75 (11) Å^3^	Prism, colorless
*Z* = 2	0.27 × 0.16 × 0.10 mm
*F*(000) = 404	

### Data collection

**Table:**

Bruker D8 Venture diffractometer	2900 independent reflections
Radiation source: fine-focus sealed tube	2454 reflections with *I* > 2σ(*I*)
Multilayered confocal mirror monochromator	*R*_int_ = 0.046
Detector resolution: 7.4074 pixels mm^-1^	θ_max_ = 25.1°, θ_min_ = 2.4°
φ and ω scans	*h* = −11→11
Absorption correction: multi-scan (*SADABS*; Bruker, 2016)	*k* = −6→7
*T*_min_ = 0.97, *T*_max_ = 0.99	*l* = −20→20
8906 measured reflections	

### Refinement

**Table:**

Refinement on *F*^2^	Primary atom site location: structure-invariant direct methods
Least-squares matrix: full	Secondary atom site location: difference Fourier map
*R*[*F*^2^ > 2σ(*F*^2^)] = 0.046	Hydrogen site location: inferred from neighbouring sites
*wR*(*F*^2^) = 0.099	H-atom parameters constrained
*S* = 1.07	*w* = 1/[σ^2^(*F*_o_^2^) + 1.1579*P*] where *P* = (*F*_o_^2^ + 2*F*_c_^2^)/3
2900 reflections	(Δ/σ)_max_ < 0.001
250 parameters	Δρ_max_ = 0.25 e Å^−^^3^
1 restraint	Δρ_min_ = −0.25 e Å^−^^3^

### Special details

**Table:**

Experimental. IR (film): 3423, 2985, 2934, 1631, 1488, 1245, 1039 cm^-1^; ^1^H NMR (500 MHz, CDCl_3_): *δ* (p.p.m.) 6.74 (d, *J* = 8.0 Hz, 1H), 6.70 (d, *J* = 1.7 Hz, 1H), 6.67 (dd, *J* = 8.0, 1.7 Hz, 1H), 5.92 (s, 2H), 5.91 (ddd, *J* = 15.5, 7.7, 0.9 Hz, 1H), 5.59 (ddd, *J* = 15.5, 8.0, 0.9 Hz, 1H), 4.65 (ddd, *J* = 8.0, 6.6, 0.9 Hz, 1H), 4.21 (ddd, *J* = 7.8, 6.6, 4.9 Hz, 1H), 3.87 (dddd, *J* = 8.3, 7.7, 6.3, 0.9 Hz, 1H), 3.65 (dd, *J* = 11.5, 7.8 Hz, 1H), 3.53 (dd, *J* = 11.5, 4.9 Hz, 1H), 3.05 (bs, 1H), 2.97 (s, 3H), 2.92 (s, 3H), 2.69 (dd, *J* = 15.8, 8.3 Hz, 1H), 2.65 (dd, *J* = 15.8, 6.3 Hz, 1H), 1.44 (s, 3H), 1.34 (s, 3H) ^13^C NMR (125 MHz, CDCl_3_): *δ* (p.p.m.) 171.5 (C), 147.9 (C), 146.3 (C), 137.2 (CH), 137.0 (C), 125.7 (CH), 120.4 (CH), 108.5 (CH), 108.5 (C), 108.0 (CH), 101.1 (CH_2_), 78.5 (CH), 78.0 (CH), 61.2 (CH_2_), 44.3 (CH), 39.5 (CH_2_), 37.4 (CH_3_), 35.7 (CH_3_), 27.9 (CH_3_), 25.2 (CH_3_)
Geometry. All esds (except the esd in the dihedral angle between two l.s. planes) are estimated using the full covariance matrix. The cell esds are taken into account individually in the estimation of esds in distances, angles and torsion angles; correlations between esds in cell parameters are only used when they are defined by crystal symmetry. An approximate (isotropic) treatment of cell esds is used for estimating esds involving l.s. planes.
Refinement. Refinement of *F*^2^ against ALL reflections. The weighted *R*-factor *wR* and goodness of fit *S* are based on *F*^2^, conventional *R*-factors *R* are based on *F*, with *F* set to zero for negative *F*^2^. The threshold expression of *F*^2^ > 2*σ*(*F*^2^) is used only for calculating *R*-factors(gt) *etc.* and is not relevant to the choice of reflections for refinement. *R*-factors based on *F*^2^ are statistically about twice as large as those based on *F*, and *R*-factors based on ALL data will be even larger.

### Fractional atomic coordinates and isotropic or equivalent isotropic displacement parameters (Å^2^)

**Table:**

	*x*	*y*	*z*	*U*_iso_*/*U*_eq_	Occ. (<1)
C1	0.7840 (5)	0.4010 (8)	0.3009 (2)	0.0229 (11)	
O2	0.7683 (3)	0.1982 (5)	0.30040 (17)	0.0241 (8)	
N3	0.9032 (4)	0.4967 (7)	0.2740 (2)	0.0264 (10)	
C4	1.0102 (5)	0.3627 (10)	0.2343 (3)	0.0399 (14)	
H4A	1.1059	0.3872	0.2585	0.06*	0.54 (8)
H4B	1.0119	0.4047	0.1791	0.06*	0.54 (8)
H4C	0.9845	0.2065	0.2386	0.06*	0.54 (8)
H4D	1.0776	0.4595	0.2074	0.06*	0.46 (8)
H4E	0.961	0.2664	0.1961	0.06*	0.46 (8)
H4F	1.0637	0.2726	0.2726	0.06*	0.46 (8)
C5	0.9320 (5)	0.7318 (9)	0.2772 (3)	0.0392 (14)	
H5A	0.9038	0.7994	0.2272	0.059*	
H5B	1.0353	0.7564	0.2877	0.059*	
H5C	0.8762	0.7985	0.3189	0.059*	
C6	0.6671 (5)	0.5503 (8)	0.3324 (3)	0.0231 (11)	
H6A	0.6536	0.678	0.297	0.028*	
H6B	0.6991	0.6073	0.3841	0.028*	
C7	0.5221 (5)	0.4312 (8)	0.3404 (2)	0.0203 (11)	
H7	0.5401	0.2945	0.3718	0.024*	
C8	0.4522 (4)	0.3634 (8)	0.2637 (2)	0.0196 (10)	
H8	0.3636	0.285	0.2667	0.024*	
C9	0.4993 (5)	0.3999 (8)	0.1930 (2)	0.0196 (10)	
H9	0.5862	0.4819	0.1887	0.024*	
C10	0.4268 (5)	0.3224 (8)	0.1190 (2)	0.0226 (11)	
H10	0.411	0.4503	0.0829	0.027*	
O11	0.2905 (3)	0.2221 (6)	0.13541 (17)	0.0267 (8)	
C12	0.2523 (5)	0.0853 (9)	0.0707 (3)	0.0293 (12)	
O13	0.3853 (3)	0.0390 (6)	0.03211 (17)	0.0307 (8)	
C14	0.5029 (5)	0.1354 (8)	0.0758 (3)	0.0226 (11)	
H14	0.5745	0.1988	0.0393	0.027*	
C15	0.5753 (5)	−0.0380 (8)	0.1267 (3)	0.0285 (12)	
H15A	0.5174	−0.0624	0.1737	0.034*	
H15B	0.5794	−0.1789	0.0977	0.034*	
O16	0.7180 (4)	0.0271 (7)	0.14982 (19)	0.0412 (10)	
H16	0.7171	0.0809	0.195	0.062*	
C17	0.1518 (5)	0.2026 (10)	0.0138 (3)	0.0397 (14)	
H17A	0.0589	0.2292	0.0383	0.06*	
H17B	0.1365	0.1109	−0.0327	0.06*	
H17C	0.1946	0.3437	−0.0011	0.06*	
C18	0.1877 (6)	−0.1273 (10)	0.1010 (3)	0.0411 (14)	
H18A	0.2555	−0.1955	0.1387	0.062*	
H18B	0.1695	−0.2286	0.0573	0.062*	
H18C	0.0966	−0.0949	0.1265	0.062*	
C19	0.3611 (5)	0.7677 (8)	0.3512 (3)	0.0244 (11)	
H19	0.3863	0.8103	0.3	0.029*	
C20	0.4189 (4)	0.5773 (8)	0.3858 (2)	0.0193 (10)	
C21	0.3817 (4)	0.5197 (8)	0.4614 (2)	0.0217 (11)	
H21	0.4223	0.3907	0.4845	0.026*	
C22	0.2856 (5)	0.6487 (8)	0.5039 (3)	0.0262 (12)	
H22	0.2598	0.6091	0.5554	0.031*	
C23	0.2304 (4)	0.8334 (9)	0.4685 (2)	0.0223 (11)	
C24	0.2668 (5)	0.8908 (8)	0.3937 (3)	0.0248 (11)	
O25	0.1991 (3)	1.0850 (6)	0.37196 (19)	0.0325 (9)	
C26	0.0979 (5)	1.1294 (9)	0.4323 (3)	0.0329 (13)	
H26A	−0.0019	1.0976	0.4134	0.039*	
H26B	0.1034	1.2863	0.4481	0.039*	
O27	0.1358 (4)	0.9890 (6)	0.49743 (18)	0.0340 (9)	

### Atomic displacement parameters (Å^2^)

**Table:**

	*U*^11^	*U*^22^	*U*^33^	*U*^12^	*U*^13^	*U*^23^
C1	0.024 (3)	0.029 (3)	0.016 (2)	0.004 (2)	−0.0004 (19)	−0.002 (2)
O2	0.0264 (18)	0.019 (2)	0.0266 (18)	0.0065 (16)	0.0026 (14)	0.0004 (15)
N3	0.020 (2)	0.021 (2)	0.039 (2)	0.0047 (19)	0.0074 (18)	0.002 (2)
C4	0.026 (3)	0.040 (4)	0.054 (3)	0.010 (3)	0.013 (3)	0.004 (3)
C5	0.023 (3)	0.032 (3)	0.063 (4)	−0.003 (3)	0.006 (3)	0.000 (3)
C6	0.026 (2)	0.021 (3)	0.022 (2)	0.002 (2)	0.0007 (19)	−0.003 (2)
C7	0.020 (2)	0.019 (3)	0.022 (2)	0.003 (2)	0.0036 (19)	−0.002 (2)
C8	0.016 (2)	0.017 (3)	0.026 (2)	0.002 (2)	−0.0001 (19)	−0.004 (2)
C9	0.020 (2)	0.016 (3)	0.023 (2)	0.001 (2)	0.0035 (19)	−0.004 (2)
C10	0.025 (3)	0.022 (3)	0.021 (2)	0.001 (2)	0.0038 (19)	0.003 (2)
O11	0.0209 (17)	0.033 (2)	0.0267 (18)	−0.0023 (16)	0.0037 (13)	−0.0100 (16)
C12	0.029 (3)	0.034 (3)	0.025 (3)	0.000 (3)	0.002 (2)	−0.009 (2)
O13	0.0283 (18)	0.035 (2)	0.0285 (18)	−0.0009 (17)	−0.0008 (14)	−0.0128 (17)
C14	0.025 (3)	0.023 (3)	0.020 (2)	0.000 (2)	0.0027 (19)	−0.004 (2)
C15	0.034 (3)	0.024 (3)	0.028 (3)	0.005 (2)	−0.002 (2)	−0.006 (2)
O16	0.034 (2)	0.052 (3)	0.037 (2)	0.014 (2)	−0.0049 (15)	−0.015 (2)
C17	0.035 (3)	0.050 (4)	0.034 (3)	0.007 (3)	−0.008 (2)	−0.009 (3)
C18	0.038 (3)	0.041 (4)	0.044 (3)	−0.007 (3)	−0.001 (3)	−0.001 (3)
C19	0.025 (2)	0.028 (3)	0.021 (2)	−0.001 (2)	0.0068 (19)	0.001 (2)
C20	0.018 (2)	0.025 (3)	0.015 (2)	0.000 (2)	0.0010 (18)	−0.004 (2)
C21	0.019 (2)	0.021 (3)	0.025 (2)	−0.001 (2)	−0.0010 (19)	−0.002 (2)
C22	0.026 (3)	0.033 (3)	0.020 (2)	−0.002 (2)	0.006 (2)	−0.003 (2)
C23	0.016 (2)	0.031 (3)	0.021 (2)	−0.007 (2)	0.0101 (19)	−0.012 (2)
C24	0.020 (2)	0.025 (3)	0.029 (3)	0.001 (2)	0.001 (2)	−0.002 (2)
O25	0.0294 (19)	0.026 (2)	0.043 (2)	0.0087 (17)	0.0114 (15)	0.0002 (17)
C26	0.027 (3)	0.031 (3)	0.040 (3)	0.000 (2)	0.010 (2)	−0.014 (3)
O27	0.0312 (19)	0.038 (2)	0.033 (2)	0.0068 (18)	0.0115 (15)	−0.0093 (18)

### Geometric parameters (Å, º)

**Table:**

C1—O2	1.239 (6)	C12—C18	1.518 (7)
C1—N3	1.339 (6)	O13—C14	1.429 (5)
C1—C6	1.521 (6)	C14—C15	1.512 (6)
N3—C5	1.452 (7)	C14—H14	1.0
N3—C4	1.462 (6)	C15—O16	1.425 (6)
C4—H4A	0.98	C15—H15A	0.99
C4—H4B	0.98	C15—H15B	0.99
C4—H4C	0.98	O16—H16	0.84
C4—H4D	0.98	C17—H17A	0.98
C4—H4E	0.98	C17—H17B	0.98
C4—H4F	0.98	C17—H17C	0.98
C5—H5A	0.98	C18—H18A	0.98
C5—H5B	0.98	C18—H18B	0.98
C5—H5C	0.98	C18—H18C	0.98
C6—C7	1.533 (6)	C19—C24	1.373 (6)
C6—H6A	0.99	C19—C20	1.398 (7)
C6—H6B	0.99	C19—H19	0.95
C7—C8	1.508 (6)	C20—C21	1.394 (6)
C7—C20	1.530 (6)	C21—C22	1.403 (6)
C7—H7	1.0	C21—H21	0.95
C8—C9	1.317 (6)	C22—C23	1.366 (7)
C8—H8	0.95	C22—H22	0.95
C9—C10	1.496 (6)	C23—C24	1.379 (6)
C9—H9	0.95	C23—O27	1.387 (6)
C10—O11	1.435 (5)	C24—O25	1.381 (6)
C10—C14	1.535 (6)	O25—C26	1.439 (5)
C10—H10	1.0	C26—O27	1.440 (6)
O11—C12	1.422 (5)	C26—H26A	0.99
C12—O13	1.440 (5)	C26—H26B	0.99
C12—C17	1.509 (7)		
			
O2—C1—N3	121.7 (4)	C17—C12—C18	112.3 (4)
O2—C1—C6	120.6 (4)	C14—O13—C12	109.1 (3)
N3—C1—C6	117.7 (4)	O13—C14—C15	109.8 (4)
C1—N3—C5	124.4 (4)	O13—C14—C10	101.7 (3)
C1—N3—C4	119.6 (4)	C15—C14—C10	115.9 (4)
C5—N3—C4	116.0 (4)	O13—C14—H14	109.7
N3—C4—H4A	109.5	C15—C14—H14	109.7
N3—C4—H4B	109.5	C10—C14—H14	109.7
H4A—C4—H4B	109.5	O16—C15—C14	111.2 (4)
N3—C4—H4C	109.5	O16—C15—H15A	109.4
H4A—C4—H4C	109.5	C14—C15—H15A	109.4
H4B—C4—H4C	109.5	O16—C15—H15B	109.4
N3—C4—H4D	109.5	C14—C15—H15B	109.4
N3—C4—H4E	109.5	H15A—C15—H15B	108.0
H4D—C4—H4E	109.5	C15—O16—H16	109.5
N3—C4—H4F	109.5	C12—C17—H17A	109.5
H4D—C4—H4F	109.5	C12—C17—H17B	109.5
H4E—C4—H4F	109.5	H17A—C17—H17B	109.5
N3—C5—H5A	109.5	C12—C17—H17C	109.5
N3—C5—H5B	109.5	H17A—C17—H17C	109.5
H5A—C5—H5B	109.5	H17B—C17—H17C	109.5
N3—C5—H5C	109.5	C12—C18—H18A	109.5
H5A—C5—H5C	109.5	C12—C18—H18B	109.5
H5B—C5—H5C	109.5	H18A—C18—H18B	109.5
C1—C6—C7	112.6 (4)	C12—C18—H18C	109.5
C1—C6—H6A	109.1	H18A—C18—H18C	109.5
C7—C6—H6A	109.1	H18B—C18—H18C	109.5
C1—C6—H6B	109.1	C24—C19—C20	117.7 (4)
C7—C6—H6B	109.1	C24—C19—H19	121.1
H6A—C6—H6B	107.8	C20—C19—H19	121.1
C8—C7—C20	110.0 (3)	C21—C20—C19	120.1 (4)
C8—C7—C6	114.0 (4)	C21—C20—C7	120.0 (4)
C20—C7—C6	109.4 (4)	C19—C20—C7	120.0 (4)
C8—C7—H7	107.7	C20—C21—C22	121.2 (4)
C20—C7—H7	107.7	C20—C21—H21	119.4
C6—C7—H7	107.7	C22—C21—H21	119.4
C9—C8—C7	127.7 (4)	C23—C22—C21	117.4 (4)
C9—C8—H8	116.1	C23—C22—H22	121.3
C7—C8—H8	116.1	C21—C22—H22	121.3
C8—C9—C10	125.1 (4)	C22—C23—C24	121.5 (4)
C8—C9—H9	117.4	C22—C23—O27	129.1 (4)
C10—C9—H9	117.4	C24—C23—O27	109.4 (4)
O11—C10—C9	110.1 (4)	C19—C24—C23	122.1 (5)
O11—C10—C14	101.4 (4)	C19—C24—O25	127.6 (4)
C9—C10—C14	116.1 (4)	C23—C24—O25	110.3 (4)
O11—C10—H10	109.6	C24—O25—C26	105.3 (4)
C9—C10—H10	109.6	O25—C26—O27	107.3 (4)
C14—C10—H10	109.6	O25—C26—H26A	110.3
C12—O11—C10	107.2 (3)	O27—C26—H26A	110.3
O11—C12—O13	105.9 (3)	O25—C26—H26B	110.3
O11—C12—C17	111.5 (4)	O27—C26—H26B	110.3
O13—C12—C17	108.3 (4)	H26A—C26—H26B	108.5
O11—C12—C18	108.7 (4)	C23—O27—C26	105.6 (3)
O13—C12—C18	109.9 (4)		
			
O2—C1—N3—C5	−176.1 (5)	C9—C10—C14—C15	36.9 (6)
C6—C1—N3—C5	3.8 (7)	O13—C14—C15—O16	159.9 (3)
O2—C1—N3—C4	7.7 (7)	C10—C14—C15—O16	−85.6 (5)
C6—C1—N3—C4	−172.4 (4)	C24—C19—C20—C21	−0.8 (6)
O2—C1—C6—C7	−15.6 (6)	C24—C19—C20—C7	178.7 (4)
N3—C1—C6—C7	164.4 (4)	C8—C7—C20—C21	124.5 (4)
C1—C6—C7—C8	−66.5 (5)	C6—C7—C20—C21	−109.5 (5)
C1—C6—C7—C20	169.8 (4)	C8—C7—C20—C19	−55.0 (6)
C20—C7—C8—C9	121.4 (5)	C6—C7—C20—C19	71.0 (5)
C6—C7—C8—C9	−1.9 (7)	C19—C20—C21—C22	0.6 (6)
C7—C8—C9—C10	178.2 (4)	C7—C20—C21—C22	−179.0 (4)
C8—C9—C10—O11	6.0 (7)	C20—C21—C22—C23	−0.3 (7)
C8—C9—C10—C14	−108.5 (5)	C21—C22—C23—C24	0.4 (7)
C9—C10—O11—C12	−159.5 (4)	C21—C22—C23—O27	−179.2 (4)
C14—C10—O11—C12	−36.0 (4)	C20—C19—C24—C23	0.9 (7)
C10—O11—C12—O13	21.5 (5)	C20—C19—C24—O25	178.9 (4)
C10—O11—C12—C17	−96.1 (5)	C22—C23—C24—C19	−0.7 (7)
C10—O11—C12—C18	139.5 (4)	O27—C23—C24—C19	178.9 (4)
O11—C12—O13—C14	3.5 (5)	C22—C23—C24—O25	−179.0 (4)
C17—C12—O13—C14	123.2 (4)	O27—C23—C24—O25	0.6 (5)
C18—C12—O13—C14	−113.8 (4)	C19—C24—O25—C26	172.4 (5)
C12—O13—C14—C15	98.5 (4)	C23—C24—O25—C26	−9.4 (5)
C12—O13—C14—C10	−24.8 (5)	C24—O25—C26—O27	14.4 (5)
O11—C10—C14—O13	36.5 (4)	C22—C23—O27—C26	−172.0 (5)
C9—C10—C14—O13	155.8 (4)	C24—C23—O27—C26	8.4 (5)
O11—C10—C14—C15	−82.5 (4)	O25—C26—O27—C23	−14.1 (5)

### Hydrogen-bond geometry (Å, º)

*Cg* is the centroid of the C19–C24 benzene ring.

**Table:**

*D*—H···*A*	*D*—H	H···*A*	*D*···*A*	*D*—H···*A*
O16—H16···O2	0.84	1.99	2.810 (5)	166
C14—H14···O13^i^	1.00	2.43	3.253 (6)	139
C5—H5*A*···O16^ii^	0.98	2.55	3.417 (7)	147
C22—H22···O2^iii^	0.95	2.55	3.417 (5)	152
C21—H21···*Cg*^iv^	0.95	2.98	3.794 (4)	145

Symmetry codes: (i) −*x*+1, *y*+1/2, −*z*; (ii) *x*, *y*+1, *z*; (iii) −*x*+1, *y*+1/2, −*z*+1; (iv) −*x*+1, *y*−1/2, −*z*+1.
